# Fatigue in primary Sjögren's syndrome is associated with lower levels of proinflammatory cytokines

**DOI:** 10.1136/rmdopen-2016-000282

**Published:** 2016-07-19

**Authors:** Nadia Howard Tripp, Jessica Tarn, Andini Natasari, Colin Gillespie, Sheryl Mitchell, Katie L Hackett, Simon J Bowman, Elizabeth Price, Colin T Pease, Paul Emery, Peter Lanyon, John Hunter, Monica Gupta, Michele Bombardieri, Nurhan Sutcliffe, Costantino Pitzalis, John McLaren, Annie Cooper, Marian Regan, Ian Giles, David A Isenberg, Vadivelu Saravanan, David Coady, Bhaskar Dasgupta, Neil McHugh, Steven Young-Min, Robert Moots, Nagui Gendi, Mohammed Akil, Bridget Griffiths, Dennis W Lendrem, Wan-Fai Ng

**Affiliations:** 1 Musculoskeletal Research Group, Institute of Cellular Medicine, Newcastle University, Newcastle-upon-Tyne, UK; 2 Newcastle-upon-Tyne NHS Foundation Trust, Newcastle-upon-Tyne, UK; 3 Department of Mathematics and Statistics, Newcastle University, Newcastle-upon-Tyne, UK; 4 University Hospital Birmingham, Birmingham, UK; 5 Great Western Hospitals NHS Foundation Trust, Swindon, UK; 6 Section of Musculoskeletal Disease, NIHR Leeds Musculoskeletal Biomedical Research Unit, Leeds Institute of Molecular Medicine, University of Leeds, Leeds Teaching Hospitals Trust, Leeds, UK; 7 Nottingham University Hospitals NHS Trust, Nottingham, UK; 8 Gartnavel General Hospital, Glasgow, UK; 9 Barts Health NHS Trust & Barts and the London School of Medicine & Dentistry, London, UK; 10 NHS Fife, Whyteman's Brae Hospital, Kirkaldy, UK; 11 Royal Hampshire County Hospital, Winchester, UK; 12 Royal Derby Hospital, Derby, UK; 13 University College London Hospitals NHS Foundation Trust, London, UK; 14 Queen Elizabeth Hospital, Gateshead, UK; 15 Sunderland Royal Hospital, Sunderland, UK; 16 Southend University Hospital, Westcliff-on-sea, UK; 17 Royal National Hospital for Rheumatic Diseases, Bath, UK; 18 Portsmouth Hospitals NHS Trust, Portsmouth, UK; 19 Aintree University Hospitals, Liverpool, UK; 20 Basildon Hospital, Basildon, UK; 21 Royal Hallamshire Hospital, Sheffield, UK

**Keywords:** Sjøgren's Syndrome, Inflammation, Cytokines, Autoimmune Diseases

## Abstract

**Objectives:**

This article reports relationships between serum cytokine levels and patient-reported levels of fatigue, in the chronic immunological condition primary Sjögren's syndrome (pSS).

**Methods:**

Blood levels of 24 cytokines were measured in 159 patients with pSS from the United Kingdom Primary Sjögren's Syndrome Registry and 28 healthy non-fatigued controls. Differences between cytokines in cases and controls were evaluated using Wilcoxon test. Patient-reported scores for fatigue were evaluated, classified according to severity and compared with cytokine levels using analysis of variance. Logistic regression was used to determine the most important predictors of fatigue levels.

**Results:**

14 cytokines were significantly higher in patients with pSS (n=159) compared to non-fatigued healthy controls (n=28). While serum levels were elevated in patients with pSS compared to healthy controls, unexpectedly, the levels of 4 proinflammatory cytokines—interferon-γ-induced protein-10 (IP-10) (p=0.019), tumour necrosis factor-α (p=0.046), lymphotoxin-α (p=0.034) and interferon-γ (IFN-γ) (p=0.022)—were inversely related to patient-reported levels of fatigue. A regression model predicting fatigue levels in pSS based on cytokine levels, disease-specific and clinical parameters, as well as anxiety, pain and depression, revealed IP-10, IFN-γ (both inversely), pain and depression (both positively) as the most important predictors of fatigue. This model correctly predicts fatigue levels with reasonable (67%) accuracy.

**Conclusions:**

Cytokines, pain and depression appear to be the most powerful predictors of fatigue in pSS. Our data challenge the notion that proinflammatory cytokines directly mediate fatigue in chronic immunological conditions. Instead, we hypothesise that mechanisms regulating inflammatory responses may be important.

**Key messages**

**What is already known about this subject?**

- ‘Sickness behaviour’ describes a range of symptoms, characterised by fatigue and mediated by proinflammatory cytokines, which occur in mice after injection of lipopolysaccharide, and provides an animal model of acute fatigue within the context of infection or a proinflammatory state.
- However, inflammation does not necessarily correlate with fatigue in a number of autoimmune conditions, suggesting that inflammation may not be a direct mechanism behind persistent fatigue within the context of chronic conditions.

**What does this study add?**

- The finding that certain proinflammatory cytokines decrease as patient-reported fatigue increases in primary Sjögren's syndrome (pSS) is a novel finding.
- This may improve understanding of biological basis of fatigue and help to direct future fatigue research towards investigating dysregulation of inflammation rather than inflammation itself.

**How might this impact on clinical practice?**

- This may help to explain why levels of inflammation do not appear to correlate with patient-reported fatigue levels within the pSS population, and why treating inflammation does not necessarily improve fatigue in patients with chronic inflammatory conditions such as pSS.

## Introduction

Fatigue is a significant and debilitating symptom affecting 25% of the general population resulting in considerable morbidity and economic cost.^1–3^ It is a key feature of numerous chronic diseases, being particularly prominent in many rheumatological conditions including primary Sjögren's syndrome (pSS).^4^ ^5^

Although the biological basis of fatigue is unclear, it has been suggested that proinflammatory mechanisms play a central role, since fatigue is seen in a number of conditions with underlying immune dysregulation, and is a well-documented postinfective symptom.^6–8^ This was first suggested by a constellation of symptoms, characterised by fatigue and termed ‘sickness behaviour’, seen in mice after injection of lipopolysaccharide.^9^ Sickness behaviour is considered as an evolutionarily adaptive behavioural response to infection facilitating speedy recovery, minimising energy-expenditure and reducing environmental risks when an organism is in a weakened state during and following an infection. It is mediated by proinflammatory cytokines, thus supporting inflammation as a central component in the pathophysiology of fatigue.^8^ ^10^ In particular, recent research has focused on the role of proinflammatory cytokines in mediating fatigue,^11–14^ particularly in the context of chronic fatigue syndrome (CFS). However, levels of inflammation in some rheumatological diseases, such as rheumatoid arthritis (RA), systemic lupus erythematosus (SLE) and pSS, do not necessarily correlate with fatigue scores, suggesting that there may be a complex range of positive and negative feedback loops contributing to fatigue in autoimmune conditions.^15^ ^16^

PSS is a useful disease model for research into the biological basis of fatigue. It has clear diagnostic criteria providing a well-defined patient group, in whom immunosuppressive medications—potentially altering immune and inflammatory processes—are less commonly used compared with patients with other autoimmune diseases. There is also wide variability between patients with pSS in terms of the fatigue they experience. In pSS, fatigue does not appear to correlate well with systemic or glandular disease activity, suggesting that there may be separate pathophysiological mechanisms for fatigue and disease activity.^15^

Other studies, in pSS as well as in rheumatological diseases such as RA and SLE, have shown that measures of fatigue are not associated with markers of inflammation and disease activity scores.^17–20^ However, such studies have not examined such a range of cytokines, making our study unique. In addition to this, patients in such studies with RA and SLE usually take a number of disease-modifying or immune-suppressive medications, which could affect their inflammatory profiles, unlike patients with pSS who are less-frequently prescribed potent immune-suppressive medications.

Using gene set enrichment analysis of gene expression data from 133 patients with pSS discordant for fatigue, we have recently identified several biological pathways that are discordant between fatigued and non-fatigued patients with pSS. Furthermore, using support vector machine classification, a 55-gene signature was identified, which is predictive of fatigue level. Interestingly, none of the biological pathways or the 55 genes were overtly related to inflammation.^21^ Other studies have found that pain and depression were more strongly associated with fatigue in RA and SLE than disease activity scores or inflammatory markers.^18^ ^19^ ^22^ These observations indicate that, at least in the setting of a chronic disorder, inflammatory molecules may not directly result in fatigue.

This study examines patients from the United Kingdom Primary Sjögren's Syndrome Registry (UKPSSR).^23^ This registry consists of a large cohort of clinically well-characterised patients with pSS and matched controls. We have used UKPSSR data here to attempt to determine whether there is a relationship between serum cytokine levels and patient-reported levels of fatigue. We hypothesise that there will be a significant difference in serum cytokine levels between cases with pSS and controls, and between the higher and lower fatigue scores within the pSS patient group. We also aimed to determine important predictors of fatigue in pSS to initiate further investigation of these factors.

## Methods

### Experimental design

The objective of this study was to analyse cytokine and fatigue levels in patients with pSS in order to determine whether there is a relationship between cytokines and fatigue in pSS. We also used clinical and biological data to ascertain the most important predictors of fatigue within this patient group. Cytokine profiles were compared to healthy non-fatigued controls to examine differences between these populations. This was a case–control study using results from analysis of serum samples from a patient registry along with clinical data collected contemporaneously at the time of recruitment onto the patient registry.

### Study population

Patients were selected from the UKPSSR (http://www.sjogrensregistry.org), which holds detailed clinical, laboratory and demographic data on over 700 patients with pSS across 30 centres in the UK.^23^ All patients on UKPSSR fulfil American European Consensus Group criteria for classification of pSS. This study selected 159 female patients with pSS who displayed a range of different fatigue scores. Twenty-eight non-fatigued healthy controls from the UKPSSR were also selected. The North West Research Ethics Committee granted research ethics approval for this study. Clinical and laboratory data were collected prospectively using a standardised proforma at the time of recruitment onto the UKPSSR.

### Clinical variables and outcomes

Fatigue severity was measured using the Profile of Fatigue Questionnaire, which is validated for use in pSS.^24^ Physical fatigue was scored on a scale of 0–7 to classify patients into minimal (0–1), mild (2–3), moderate (4–5) and severe (6–7) fatigue groups based on quartile scores. People from the healthy control group were screened for the presence of fatigue using a self-completed questionnaire. None of the controls reported the presence of fatigue, sicca symptoms or other autoimmune conditions. Anxiety and depression were measured using the Hospital Anxiety and Depression Score.^25^

Other clinical parameters included systemic disease activity using the EULAR Sjögren's Syndrome Disease Activity Index (ESSDAI) and EULAR Sjögren's Syndrome Patient Reported Index (ESSPRI), as well as glandular manifestations using Schirmer's test, unstimulated oral salivary flow test and EULAR Sicca Score—a measure of overall dryness experienced by the patient.^26^ ^27^

The UKPSSR holds biobanked serum samples for each patient with pSS, which were analysed with cytometric bead array-based immunoassay allowing multiple analyses of a single sample. The following 24 cytokines were tested: cluster of differentiation 40 ligand (CD40L), cluster of differentiation 54 (CD54), cluster of differentiation 106 (CD106), E-selectin, interferon-α (IFN-α), interferon-γ (IFN-γ), interferon-γ-induced protein-10 (IP-10), interleukin-1β (IL-1β), interleukin-4 (IL-4), interleukin-6 (IL-6), interleukin-8 (IL-8), interleukin-10 (IL-10), interleukin-12p70 (IL-12p70), interleukin-12–interleukin-23p40 (IL-12/IL23-p40), interleukin-17 (IL-17), interleukin-21 (IL-21), lymphotoxin-α (LT-α), macrophage inflammatory protein 1α (MIP1α), macrophage inflammatory protein 1β (MIP-1β), monocyte chemoattractant protein-1 (MCP-1), monokine induced by γ interferon (MIG), P-selectin, regulated on activation normal T expressed and secreted (RANTES) and tumour necrosis factor-α (TNF-α). These analytes represent a broad spectrum of proinflammatory and anti-inflammatory soluble molecules with possible links to fatigue. In addition, white cell count (WCC), lymphocytes, neutrophils, haemoglobin, erythrocyte sedimentation rate (ESR) and C-reactive protein (CRP) were measured in each sample by the NHS laboratory of the recruiting centre within a day of sample collection.

### Statistical analysis

Patient demographic data are presented using median and IQR. Clinical data are presented using mean and SD. Significance was determined using Wilcoxon test.

Cytokine data are presented as box plots using the median and IQR to report key findings. Since cytokine levels were not normally distributed, a normalising log transformation was performed prior to analysis, after which analysis of variance testing was used to examine the relationship between the levels of each cytokine analyte and the corresponding fatigue score. Spearman's rank correlation coefficient was also used to measure correlation between ungrouped (continuous) fatigue scores and cytokine levels.

Ordinal logistic regression analysis was used to model predicted fatigue level against observed fatigue level, using all cytokines. WCC, lymphocytes, neutrophils, ESR, CRP, ESSDAI scores and dryness scores, were also incorporated into this model, as well as depression, anxiety and pain scores.

All statistical tests and graphics were performed using R version 3.1.1 and SAS JMP (Version 14) Statistical Data Visualization software.^28^ ^29^

## Results

### Study population

Serum samples from 159 female patients with pSS with a range of fatigue levels and 28 healthy non-fatigued female controls from the UKPSSR were used in this study. Patients with pSS were stratified into four groups according to their fatigue levels. Patients were predominantly Caucasian in both groups; however, the mean age of healthy controls was younger than the pSS group. Demographic data of cohort are summarised in table 1.

**Table 1:** Demographic summary for control and pSS fatigue groups

	Control	Minimal (0–1)	Mild (2–3)	Moderate (4–5)	Severe (6–7)	p Value
N	28	24	44	65	26	
Mean age±SD	50±13	62±10	58±14	60±12	59±13	0.005
Caucasian (%)	100	100	95.5	95.4	96.2	ns

All participants are female. Mean age was lower in the control group while ethnicity did not vary significantly across groups.

pSS, primary Sjögren's syndrome.

### Clinical differences between pSS fatigue groups

Disease and symptom duration were not significantly different between fatigue groups (table 2). Anti-Ro/La positivity and the percentage of each group prescribed potentially immune-altering medications (eg, hydroxychloroquine or prednisolone) did not differ significantly across groups (table 2). Forty-three per cent of patients overall were prescribed an immune-altering medication and this was hydroxychloroquine in the majority of such patients (table 2). Serum IgG levels decreased with increasing fatigue (p=0.008) with the mean serum IgG levels in the groups of patients with pSS with minimal and mild fatigue being above the normal ranges (table 2). Lymphocyte counts increased (p=0.002) with increasing fatigue, but the values were within normal ranges for all pSS groups (table 2). The remaining haematological parameters did not show significant differences between fatigue groups. Anxiety, depression, pain, dryness and ESSPRI (overall symptom burden) scores all increased with increasing fatigue levels (p≤0.0001) (table 2). EULAR Sicca Score, a measure of ocular and oral dryness, also increased with increasing fatigue (p=0.004). However, there was no significant relationship between systemic disease activity (measured using ESSDAI scores) and fatigue groups (table 2).

**Table 2:** Clinical summary for pSS fatigue groups showing mean±SD for key demographics, haematological and clinical variables

Variable	Minimal	Mild	Moderate	Severe	p Value
Age (years)	62±10	58±14	60±12	59±13	ns
Disease duration (years)	5.5±5.8	6.1±5.2	7.5±6.2	9.1±7.3	ns
Symptom duration (years)	13±10	13±11	14±11	16±13	ns
BMI (kg/m^2^)	25±4.4	26±4.2	26±6.3	28±7.2	ns
% Anti-Ro/La positive	91.67	95.45	83.08	92.31	ns
% Not taking any immune-altering medications	67	59	52	50	ns
% On hydroxychloroquine	17	34	37	34	ns
% On prednisolone	8	5	6	12	ns
% On ‘other’ immune-altering medications	8	2	5	4	ns
ESSDAI	5.4±5.7	7.6±8.2	5.9±5.2	7.2±6.1	ns
ESSPRI	2.9±1.3	4.3±1.4	6.6±1.4	8.3±1.1	≤0.0001
ESSPRI pain	1.4±1.5	3.2±2.5	5.4±2.6	8±1.6	≤0.0001
ESSPRI dryness	5.6±2.7	5.5±2.2	6.9±2.6	8.1±2	≤0.0001
EULAR SS	5.3±2.5	5.6±2.5	6.8±2.5	7.8±2	0.0004
HADS anxiety (0–21)	3.7±2.4	6.5±3.5	8.6±4.4	12±4.9	≤0.0001
HADS depression (0–21)	2±1.9	4±2.8	7.4±3.5	11±2.9	≤0.0001
Hb (g/dL)	12±1.6	13±1.2	13±1.2	13±1.1	ns
WCC (×10^9^/L)	5.5±1.4	5.2±1.5	5.2±2.0	6.3±2.7	ns
Neutrophil (×10^9^/L)	3.5±1.1	3.3±1.3	3.2±1.5	3.7±2	ns
Lymphocyte (×10^9^/L)	1.4±0.6	1.3±0.5	1.4±0.6	1.9±0.9	0.002
ESR (mm/h)	39±26	33±25	27±24	24±20	ns
CRP (mg/L)	6.4±5	5±4.1	5.2±5.9	6.7±5.8	ns
IgG (mg/dL)	20±8.8	18±8	15±6.5	15±4.2	0.008

BMI, body mass index; CRP, C-reactive protein; ESSDAI, EULAR Sjögren's Syndrome Disease Activity Index; ESR, erythrocyte sedimentation rate; ESSPRI, EULAR Sjögren's Syndrome Patient Reported Index; EULAR SS, EULAR Sicca Score; HADS, Hospital Anxiety and Depression Score; Hb, haemoglobin; pSS, primary Sjögren's syndrome; WCC, white cell count.

### Cytokine differences between patients with pSS and healthy controls

As expected, many proinflammatory molecules were elevated among patients with pSS compared to healthy controls, consistent with the inflammatory nature of the condition. Specifically, CD106, IP-10, IL-17, IL-21, MIP1α, TNF-α, LT-α, MIP1β, IFN-γ, MIG, IL-6, IL-10, IL-12p70 and IL-12/IL23-p40 levels were significantly higher in patients with pSS compared with controls, with eight of these cytokines having p values of ≤0.0001 between these participant groups (table 3). None of the other serum proteins were significantly different between patients with pSS and controls.

**Table 3:** Cytokine levels in patients with pSS and healthy controls

Cytokine	Controls (n=28)	Cases with pSS (n=159)	p Value
CD54	41882.42 31954.3, 68077.5	47915.84 36203.1, 76537.1	0.2599
RANTES	19117.12 13203.8, 28251.3	21472.56 15722.97, 28255.1	0.1643
CD106	67543.20 51824.4, 75985.4	80921.58 57934.1, 96570.5	**0.0042**
IL-8	37378.14 11075.6, 311730.2	35623.48 10596.6, 374424.3	0.6929
IP-10	110.24 75.0, 167.4	342.38 226.2, 540.5	**<0.0001**
IF	1.34 0.8, 2.2	1.48 0.7, 4.5	0.1659
IL-17	1.32 0.4, 2.0	3.28 1.3, 47.0	**<0.0001**
IL-21	45.33 30.6, 63.4	71.71 40.0, 782.8	**0.0006**
MIP1α	5.85 1.7, 101.4	99.52 6.9, 219.3	**<0.0001**
TNF-α	0.08 0.0, 0.1	7.00 0.1, 27.1	**<0.0001**
LT-α	0.33 0.2, 0.6	2.5 0.5, 13.0	**<0.0001**
P-selectin	7385.86 5802.8, 8894.1	8212.16 5148.01, 11983.12	0.3287
MCP-1	131.62 95.3, 221.7	170.42 121.8, 318.2	0.1061
E-selectin	2515.06 1691.2, 3588.5	2862.34 1992.0, 4241.0	0.3213
MIP1β	78.96 27.0, 136.5	178.40 97.3, 333.8	**<0.0001**
IFN-γ	1.90 0.5, 3.2	2.97 1.4, 10.6	**0.0018**
MIG	125.90 84.5, 244.0	986.32 458.4, 2593.4	**<0.0001**
CD40 ligand	2838.26 1893.4, 3559.4	2449.40 1825.4, 3239.5	0.1065
IL-6	938.18 506.7, 1537.1	1544.46 836.5, 2931.9	**0.0020**
IL-1β	126 45.8, 698.7	271.23 47.7, 738.2	0.3767
IL-10	50.68 9.9, 360.0	490.90 129.6, 881.9	**<0.0001**
IL-12p70	16.63 8.40, 25.0	27.18 13.41, 206.4	**0.0024**
IL-4	0.00 0.00, 0.00	0.00 0.00. 0.00	0.3322
IL-12.IL-3p40	0.00 0.00, 0.00	0.00 0.00, 0.00	**0.0117**

Bold typeface indicates significance.

Generally cytokines were significantly higher in patients with pSS compared to controls. Values in table represent median and 25th, 75th centile (pmol/L).

CD, cluster of differentiation; IFN-γ, interferon-γ; IL, interleukin; IP-10, interferon-γ-induced protein-10; LT-α, lymphotoxin-α; MCP-1, monocyte chemoattractant protein-1; MIG, monokine induced by γ interferon; MIP, macrophage inflammatory protein; pSS, primary Sjögren's syndrome; RANTES, regulated on activation normal T expressed and secreted; TNF-α, tumour necrosis factor-α.

In addition to this, there were statistical differences in IP-10, IL-6, IL-10, IL-12, IL-17, IL-21, IFN-γ, LT-α, MIG, MIP1α, MIP1β and TNF-α levels between healthy controls and the minimally fatigued pSS groups. In all cases, they were higher in the pSS population.

### Cytokines and fatigue scores in patients with pSS

**Figure 1:**
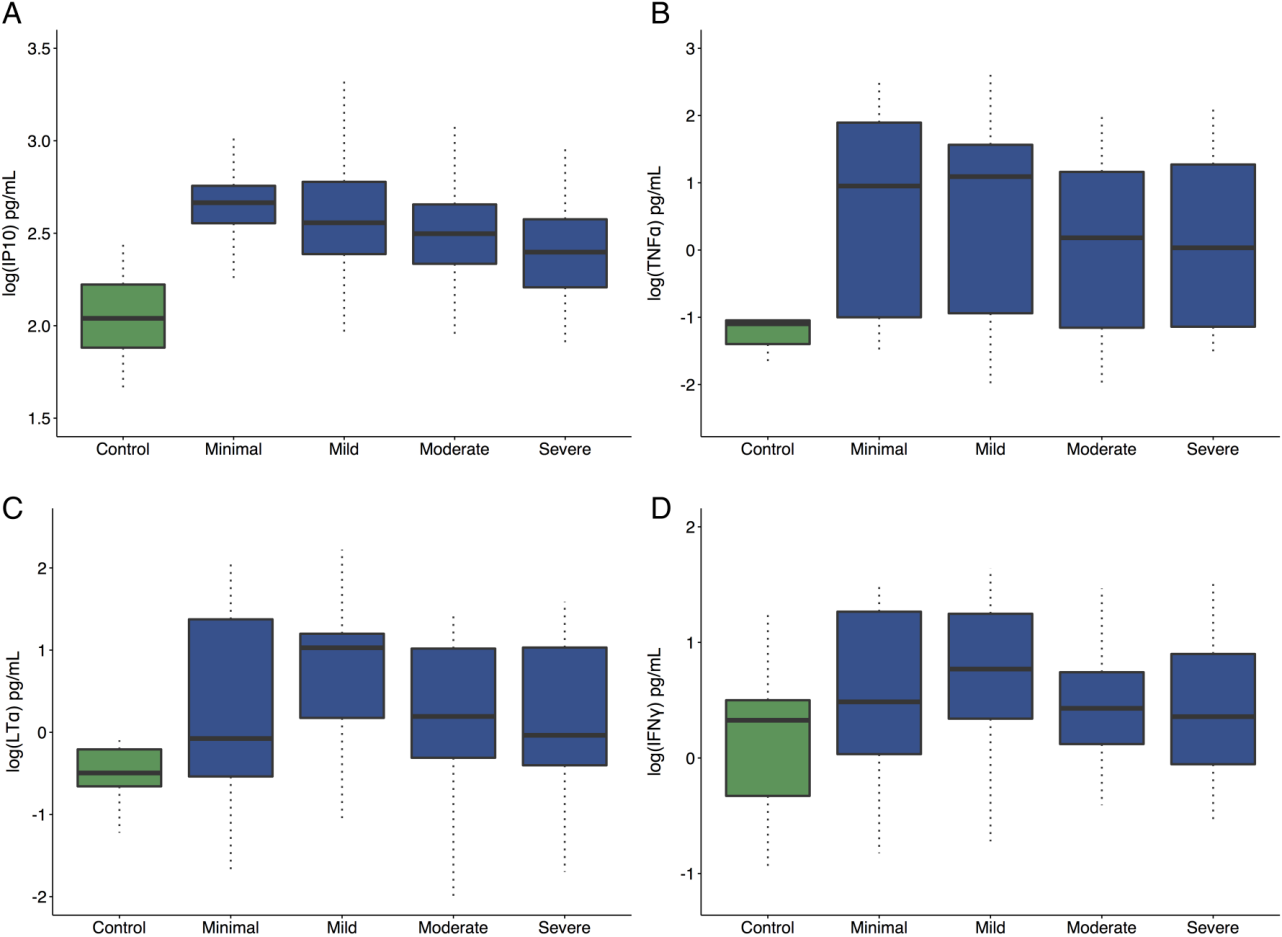
Box plot showing median cytokine levels and IQRs for (A) IP-10, (B) TNF-α, (C) LT-α and (D) IFN-γ in controls and pSS fatigue groups. In all four cases, the levels of anti-inflammatory cytokines are significantly higher in patients with pSS. In addition, all four show an inverse relationship between fatigue severity and cytokine levels. Fatigue levels fall with increasing levels of the four cytokines. IFN-γ, interferon-γ; IP-10, interferon-γ-induced protein-10; LT-α, lymphotoxin-α; pSS, primary Sjögren's syndrome; TNF-α, tumour necrosis factor-α.

Unexpectedly, fatigue levels increased with decreasing levels of several proinflammatory cytokines: IP-10 (p=0.019), TNF-α (p=0.046), LT-α (p=0.034) and IFN-γ (p=0.022) (figure 1A–D) within the cases with pSS. Furthermore, weak negative correlations were shown between cytokine levels and ungrouped (continuous) fatigue scores: IP-10—0.2190, TNF-α—0.1273, IFN-γ—0.1985 and LT-α—0.0808. The remaining cytokines did not display statistically significant relationships with fatigue levels within the cases with pSS.

### Predictors of fatigue severity in pSS

**Figure 2:**
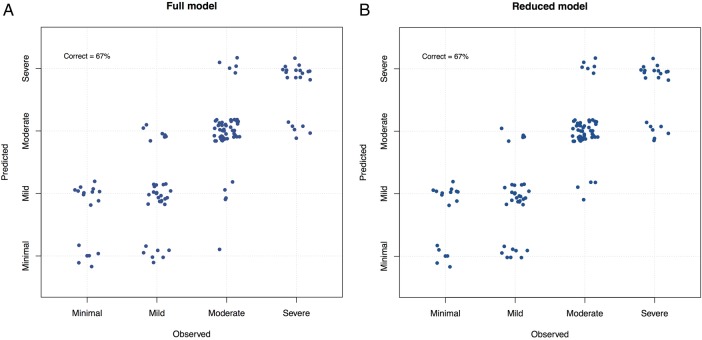
(A) Full ordinal logistic regression model with all parameters. This model analyzes observed fatigue values in order to predict fatigue values based on the following variables: all 24 cytokines, WCC, lymphocytes, neutrophils, ESR, CRP, ESSDAI scores, dryness scores, depression, pain and anxiety scores. It then compares the predicted with the observed values to ascertain the accuracy of the model. All of these variables predict fatigue level correctly in 67% of cases. (B) shows that IFN-γ, IP-10, depression and pain alone predicted fatigue level with 67% accuracy, which was as effective as the full-model.

Ordinal logistic regression (figure 2A, B) predicts membership of the minimal, mild, moderate and severe fatigue groups using all 24 cytokines, WCC, lymphocytes, neutrophils, ESR, CRP, ESSDAI scores and dryness scores, as well as patient-reported depression, anxiety and pain. The full model, with all parameters, correctly predicts fatigue in 67% of cases (figure 2A). This model with all parameters was robust to the presence or absence of loose markers of disease activity (such as WCC, lymphocytes, neutrophils, ESR, CRP, ESSDAI and dryness scores), but sensitive to the presence or absence of cytokines, depression, anxiety and pain. The model predictions are reasonably accurate providing cytokines, depression and pain are retained. This suggests that measures of disease activity in pSS appear to be less important than cytokines, depression and pain in accurately predicting fatigue levels.

Refinement of the ordinal logistic regression model, identified IFN-γ, IP-10, depression and pain are sufficient to predict fatigue with similar (67%) accuracy as the full model (figure 2B). This suggests that depression, pain and cytokines are the most important predictors of fatigue.

## Discussion

Our study demonstrates that patients with pSS with higher levels of fatigue had lower levels of the proinflammatory cytokines IP-10, TNF-α, LT-α and IFN-γ than patients with pSS with low levels of fatigue. It should be noted, however, that the serum levels of many cytokines among even the fatigued participants with pSS were still higher than non-fatigued healthy individuals. Our observation that the magnitude of inflammatory response correlated inversely with fatigue levels does not, however, support a simple concept of higher levels of inflammation leading to worse fatigue.

**Figure 3:**
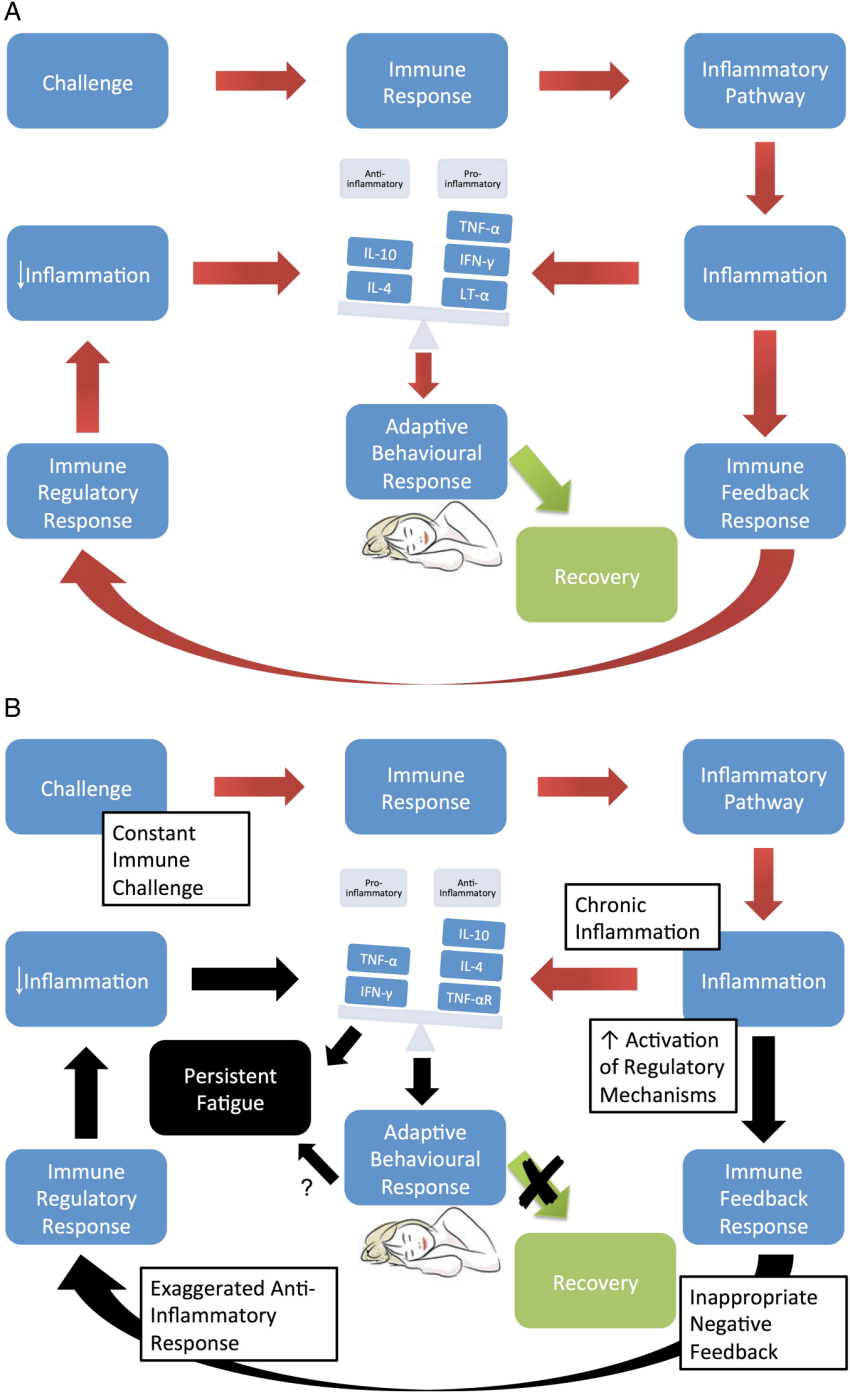
Hypothetical model of fatigue in a chronic immunological condition. This model suggests that anti-inflammatory mechanisms may have a part to play in the persistent fatigue in chronic inflammatory diseases. When presented with an immune infective challenge, the immune response triggers inflammatory pathways, which triggers a cytokine-mediated behavioural response, which has been called ‘sickness behaviour’. Additionally, the immune (inflammatory) response also activates homeostatic regulatory pathways. In the healthy patient (A), the cytokine balance is restored and the behavioural pathways inactivated, leading to recovery. However, if this system is dysregulated (B) and exposed to constant immune challenge, as in the case of pSS, chronic inflammation results, which triggers an inappropriate anti-inflammatory response. We postulate that this exaggerated immune regulatory response turns what was an adaptive behavioural response into persistent and pathological chronic fatigue. This may help to explain why studies have found raised levels of anti-inflammatory cytokines in patients with more severe fatigue and why proinflammatory cytokines decrease as fatigue increases in this study. The fatigued patient is caught in a pathological feedback loop with dysregulation of the immune system, cytokine. pSS, primary Sjögren's syndrome.

One possible explanation is that regulatory mechanisms of inflammation may be responsible for sustained fatigue after an initial inflammatory response. This hypothesis is illustrated in figure 3, which depicts an anti-inflammatory negative feedback loop, reducing inflammatory markers, but resulting in persistent fatigue. Thus, although fatigue is induced by proinflammatory cytokines as part of an ‘adaptive behaviour response’, which has evolved as a protective motivational state during and following an infection, a potentially maladaptive immune response may contribute to the maintenance of persistent fatigue after clearance of a pathogen or in a chronic inflammatory state.^30^ We suggest that immune-modulatory and anti-inflammatory mechanisms may be inappropriately expressed in patients suffering from fatigue. Further research into such anti-inflammatory pathways and regulatory mechanisms of inflammation might be insightful in the understanding of the biological basis of persistent fatigue in chronic diseases.

This hypothesis may be further supported by a recent study by Hornig *et al*,^31^ which demonstrated lower levels of the proinflammatory cytokines IL-17, IL-8, IP-10, and TNF-α and soluble Fas ligand in patients with CFS compared with control groups. This study also found inverse correlations with cytokine levels and illness duration in patients with CFS. Specifically, the proinflammatory cytokines IFN-γ and IL-12p40 were more markedly elevated in the short-duration compared to the long-duration CFS group. This suggests that the immunological changes seen in CFS are dynamic and may change with time as illness duration increases. These observations are consistent with the concept of fatigue being mediated by negative feedback/homeostatic mechanisms following an initial or sustained period of inflammation. An increased anti-inflammatory response has also been observed in a number of other CFS studies. In 2007, ter Wolbeek *et al* found higher levels of anti-inflammatory cytokines (IL-10 and IFN-γ/IL-10 ratios) and lower levels of proinflammatory cytokines (IL-6 and TNF-α) in adolescents with CFS compared to healthy controls.^32^ Reduced phytohemagglutinin-stimulated IFN-γ production by CD4+ T cells has been shown in CFS.^33^ This may support the idea that anti-inflammatory or regulatory pathways play some role in mediating persistent fatigue, after initial inflammatory insults.

It is also noteworthy that IP-10, TNF-α and IFN-γ have been implicated in the study by Hornig and colleagues as well as our current study. IFN-γ, produced by natural killer as well as T cells, helps to initiate a cellular response to infection. TNF-α is secreted as part of an acute phase reaction to mediate protective immune responses to infection. IP-10 is secreted from cells after stimulation by IFN-γ and is a chemoattractant for activated T cells to sites of inflammation.^34^ These cytokines are linked to an activated Th1 response, raising the intriguing possibility that dysregulation of Th1 responses may be linked to development of fatigue. A bias towards Th2 responses in CFS has, in fact, been demonstrated in a number of CFS studies and may be an important component in mediating fatigue.^35^

However, few cytokine abnormalities in CFS and other fatigue-associated chronic conditions have been consistently demonstrated across different studies. One possible explanation is the confounding psychosocial factors present in the patient groups in different studies. Indeed, depression and pain were important predictors of fatigue in our study, supporting the concept of a biopsychological model of fatigue. The importance of psychological factors in pSS-associated fatigue is supported by two recent studies. Karageorgas *et al*^36^ found that anxiety, depression and fibromyalgia play a major role in pSS-associated fatigue, whereas van Leeuwen *et al*^37^ demonstrated that distinct psychological profiles are differentially associated with fatigue in pSS. However, removing depression, pain and anxiety did not fully disrupt the regression model in this study. In contrast, removing cytokines disrupted the performance of the model suggesting that while pain and depression appear to play some role in fatigue, inflammatory/anti-inflammatory cascades may play a larger role. Further work is however necessary to understand this likely complex interplay and overlap between multiple psychosocial and biological factors, which may influence levels of fatigue.

Some of the strengths of this study include (1) a large, clinically well-defined patient group with clear diagnostic criteria; (2) minimal demographic variation between fatigue groups within the pSS cohort; (3) a validated fatigue measure for use in pSS and (4) extensive characterisation of the cytokine profiles.

We aimed to minimise potential gender differences in cytokine profiles by using a female study population; however, this may mean that such data may not be applicable to men with pSS or indeed fatigue. Although there were significant differences in age between healthy volunteers and cases with pSS, age differences between pSS fatigue groups were not statistically significant and it was within this pSS cohort that the analysis of fatigue and cytokine levels, and ordinal regression took place. It is also worth mentioning that pSS fatigue groups were not significantly different in terms of anti-Ro/La positivity or immune-altering medications used. This is relevant as hydroxychloroquine and prednisolone can affect a cytokine profile and therefore suggests that cytokine differences are not due to differential medication prescribing across groups.^38^

There are however limitations in this study. First, unlike a longitudinal study, the cross-sectional nature of the study does not permit within-patient comparisons. Second, cytokine levels may vary considerably within a short space of time and their presence in the blood may be influenced by multiple factors, including fatigue itself. While cytokines may indeed affect fatigue, fatigue may also affect cytokine levels as part of a two-way loop. Furthermore, there could be other possible contributing factors of fatigue among patients with pSS, which have not been taken into consideration.

To conclude, immune dysfunction or dysregulation may contribute to the development of fatigue in pSS and other chronic immunological diseases, although further characterisation of the mechanisms involved is needed. In particular, evaluation of potential negative feedback pathways inappropriately activated in patients experiencing fatigue, as well as studies examining different phenotypes and their corresponding levels of fatigue within a defined patient population, would be valuable areas for future research.
